# COMBINATIONS AND SEQUENCES OF LIFE TRANSITIONS DURING A 10 YEAR PERIOD AROUND RETIREMENT AGE

**DOI:** 10.1093/geroni/igad104.0737

**Published:** 2023-12-21

**Authors:** Björn Slaug, Erik Eriksson, Susanne Iwarsson, Maya Kylén, Frank Oswald, Anna Wanka, Karla Wazinki, Steven Schmidt

**Affiliations:** Lund University, Lund, Skane Lan, Sweden; Lund University, Lund, Skane Lan, Sweden; Lund University, Lund, Skane Lan, Sweden; Lund University, Lund, Skane Lan, Sweden; Goethe University Frankfurt, Frankfurt am Main, Hessen, Germany; Goethe University Frankfurt, Frankfurt am Main, Hessen, Germany; Goethe University Frankfurt, Frankfurt am Main, Hessen, Germany; Lund University, Lund, Skane Lan, Sweden

## Abstract

Our lives can be characterized as periods of stability, alternating with events that bring about change of both practical and psychological nature which require some life adjustment. The scope and depth of such life transitions are related to the kind of event, but also to whether it is perceived as positive or negative, occurs together with other events, addresses an entirely new topic or something that is biographically familiar, comes after a long period of stability or if it is an event in a chain of events occurring shortly after one another. Applying a quantitative approach to qualitative interview data from the Swedish German HoT-age project (N=50; mean age=69; 50% men), life transitions were analysed by means of sequential and configural frequency analysis. During an approximate 10-year period around retirement age, the participants experienced in average 3.7 life transitions and 24% of them more than five. Fifteen different kinds of transitions (retirement, relocation, divorce/separation, new partner, chronic illness etc) were recorded. Besides retirement experienced by almost all (96%), relocation (66%), becoming a grandparent (40%) and chronic illness (40%) were most frequent. The transitions significantly more often (p=0.041) occurring in combination were retirement and relocation. Those who had experienced chronic illness often had a sequence of several events preceding the illness recorded as a transition. Those who found a new partner often had a sequence of events after that. Large variation in sequences and distances between events were found. This study contributes with new knowledge on life transitions around retirement age.

